# Boosting Maize Yield and Mitigating Greenhouse Gas Emissions Through Synergistic Nitrogen and Chemical Regulation by Optimizing Roots and Developing Grains Under High-Density Planting in Northeast China

**DOI:** 10.3390/plants14203193

**Published:** 2025-10-17

**Authors:** Xiaoming Liu, Yao Meng, Lihua Xie, Yubo Hao, Yang Yu, Guoyi Lv, Yubo Jiang, Yiteng Zhang, Chunrong Qian, Wanrong Gu

**Affiliations:** 1 College of Agronomy, Northeast Agricultural University, Harbin 150030, China; liuxiaoming0913@163.com; 2 College of Biology and Agriculture, Jiamusi University, Jiamusi 154007, China; 3 Scientific Research Management Department, Heilongjiang Academy of Land Reclamation Sciences, Harbin 150038, China; mengyao830922@163.com; 4 Crop Development Research Institute, Heilongjiang Academy of Land Reclamation Sciences, Harbin 150038, China; xielh00@163.com; 5 Institute of Crop Cultivation and Tillage, Heilongjiang Academy of Agricultural Sciences, Harbin 150086, China; yubohao@haas.cn (Y.H.); yuyanghaas@163.com (Y.Y.); guoyilv@haas.cn (G.L.); jiangyubo@haas.cn (Y.J.); zyt@haas.cn (Y.Z.); qianchunrong@haas.cn (C.Q.)

**Keywords:** nitrogen fertilizer, chemical regulation, root growth, grain formation, greenhouse gas emissions

## Abstract

Increasing planting density is an effective strategy for enhancing maize (*Zea mays* L.) yield. However, high density often inhibits plant growth and dry matter accumulation. Synergistic nitrogen management and chemical regulation offer an effective approach to overcoming yield limitations under high-density conditions. A two-year field experiment with two maize cultivars under high density (90,000 plants ha^−1^), involving four nitrogen rates combined with PGR, explored their effects on root growth, yield formation, and greenhouse gas emissions. Results showed that 240 kg N ha^−1^ significantly improved root morphological characteristics (root dry weight, root volume, root surface, root length) and physiological traits (bleeding sap rate, etc.), with chemical regulation providing additional enhancements. Additionally, nitrogen application increased the maximum grain-filling rate (V_max_) and enzyme activity in grains, thereby enhancing grain weight; chemical regulation increased dry matter accumulation and its contribution to grains. Reduced nitrogen application combined with chemical regulation effectively decreased greenhouse gas emission. The highest maize yield was obtained under the application of 240 kg N ha^−1^ combined with chemical regulation, which promoted root growth and grain formation, thereby improving yield and reducing emissions. This study indicates that the cultivation practice combining nitrogen application with chemical regulation provides an optimized approach for environmentally friendly and high-yield maize cultivation under high planting density.

## 1. Introduction

Maize (*Zea mays* L.) is a globally crucial food crop essential for food security. Global maize demand is projected to increase steadily, driven by population growth and rising biofuel requirements [1]. In this context, high-density planting is widely adopted in major maize-producing regions as an effective strategy to improve yield [2,3]. However, excessively high planting density often negatively impacts plant growth and limits yield enhancement [4]. High planting density increases interplant competition, resulting in root growth inhibition that restricts both nutrient uptake and shoot growth [5]. Additionally, photosynthate synthesis and allocation are impaired, leading to preferential dry matter accumulation in vegetative organs at the expense of grain development [6]. These constraints ultimately limit maize yield potential. Therefore, developing optimized cultivation practices is essential to maintain maize productivity under high planting density.

Nitrogen fertilizer plays a vital role in regulating maize growth and yield under high planting density [7]. Its application strategy directly affects root growth, dry matter accumulation, and yield formation [8]. As the primary organ of water and nutrient uptake, root morphological characteristics (e.g., root length density, root distribution), and physiological traits (e.g., bleeding sap rate) that are directly regulated by nitrogen availability [9], nitrogen fertilizer can optimize root morphology and physiology, enhancing plant dry weight and nitrogen uptake efficiency [10]. Specifically, optimal nitrogen application increases root length density, root surface area, and root volume, thereby improving root growth [11]. Studies indicate that nitrogen application significantly improves root bleeding sap rate and photosynthate translocation, thereby promoting shoot growth [12]. Furthermore, nitrogen management is a key factor regulating maize yield formation. Grain formation primarily depends on concurrent photosynthate supply [13]. Consequently, grain yield is strongly correlated with biomass accumulation during the grain-filling period [14]. Studies indicate that nitrogen application at 160–320 kg ha^−1^ increases maize biomass by 24–28% relative to control plots, significantly enhancing grain yield [15]. Thus, nitrogen fertilizer represents a critical agronomic practice for maize yield improvement. However, inappropriate nitrogen management reduces nitrogen use efficiency, increases greenhouse gas emissions, and constrains yield improvement, thereby threatening agricultural sustainability [16]. Therefore, developing cultivation strategies that enhance yield and resource use efficiency is crucial for maximizing fertilizer productivity and achieving high-yielding, efficient, and environmentally sustainable crop production.

Plant growth regulators (PGRs) are effective substances for regulating plant growth in agricultural production [17]. They are artificially synthesized regulatory substances that exhibit physiological activities similar to those of plant endogenous hormones. By regulating endogenous phytohormone systems, PGRs enhance maize growth and metabolic efficiency, thereby improving stress resistance and yield potential [18,19]. In China, ethephon has been widely employed in maize production systems characterized by high planting density and nitrogen input, serving as a crucial measure to enhance lodging resistance [20,21]. Studies indicate that ethephon significantly improves post-silking nitrogen remobilization and nitrogen utilization efficiency [22]. Diethyl aminoethyl hexanoate (DA-6), a plant growth promoter, exerts a facilitative effect on plant growth, metabolism, and dry matter accumulation [23,24]. In recent years, the compound formulation comprising 30% DA-6 and ethephon integrates the advantages of the two components and has been widely applied in Chinese maize production [25]. Studies have shown that the 30% DA-6·ethephon formulation enhances root spatial distribution, promotes photosynthate accumulation and root nutrient supply, and coordinates root–shoot relationships, thereby playing a pivotal role in increasing maize yield [26]. Currently, most research on the 30% DA-6·ethephon formulation has focused on aboveground plant parts. The effects of plant growth regulator and nitrogen fertilizer on root growth characteristics under high planting density, as well as their subsequent impacts on yield formation and greenhouse gas emissions, remain unclear. Therefore, developing a robust root to enhance nutrient uptake capacity and coordinate dry matter partitioning is essential for improving crop yield and optimizing soil nutrient utilization, which is of great significance for establishing high-yield and high-efficiency cultivation systems under high planting density.

Therefore, a two-year field experiment was conducted under high planting density, involving varied nitrogen rates and chemical regulation treatments. The study focused on the following: (1) effects of nitrogen fertilizer and chemical regulation on maize root morphology and root bleeding sap; (2) their regulatory roles in grain formation and dry matter accumulation; and (3) their impacts on yield and greenhouse gas emissions. We hypothesized that moderate nitrogen reduction combined with chemical regulation can optimize root morphology, enhance root bleeding sap, improve the efficiency of dry matter partitioning to grains, and reduce greenhouse gas emissions, thereby realizing the synergy between high yield and ecological sustainability. The findings are expected to provide theoretical support for optimizing nitrogen management and chemical regulation technology in high-density maize production, thereby facilitating the environmentally friendly and sustainable development of maize cultivation.

## 2. Results

### 2.1. Root Morphology

**Figure 1:**
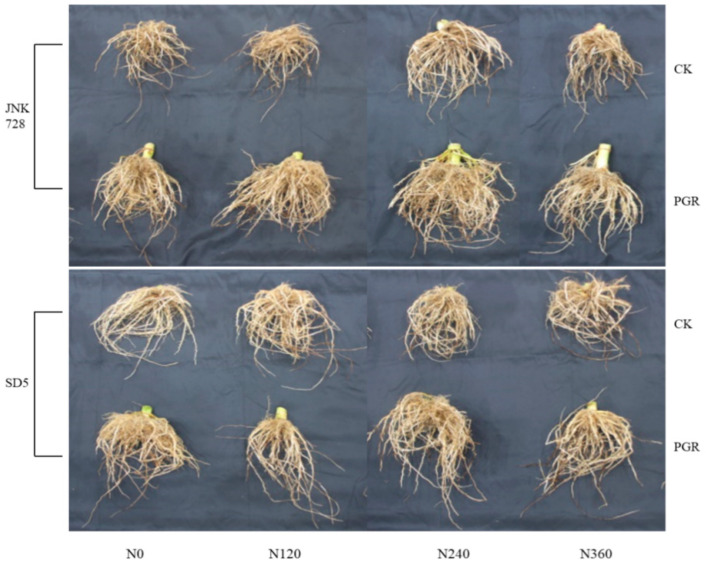
Effect of nitrogen application rates and chemical regulation on root morphology in early grain-filling stage. JNK728 and SD5 indicate maize varieties Jingnongke 728 and Saide 5, respectively. PGR and CK indicate spraying plant growth regulator and water, respectively. N0, N120, N240, and N360 indicate nitrogen application rates at 0, 120, 240, and 360 kg ha^−1^, respectively.

The root system serves as a vital absorption organ in plants, significantly influencing shoot growth and playing a crucial role in yield formation. As shown in Figure 1, both nitrogen rates and chemical regulation significantly affected root system size under high planting density. Root development initially increased then decreased with elevated nitrogen application, reaching maximum values at 240 kg N ha^−1^ (N240) treatment. Chemical regulation markedly enhanced root system expansion and promoted root growth.

**Figure 2:**
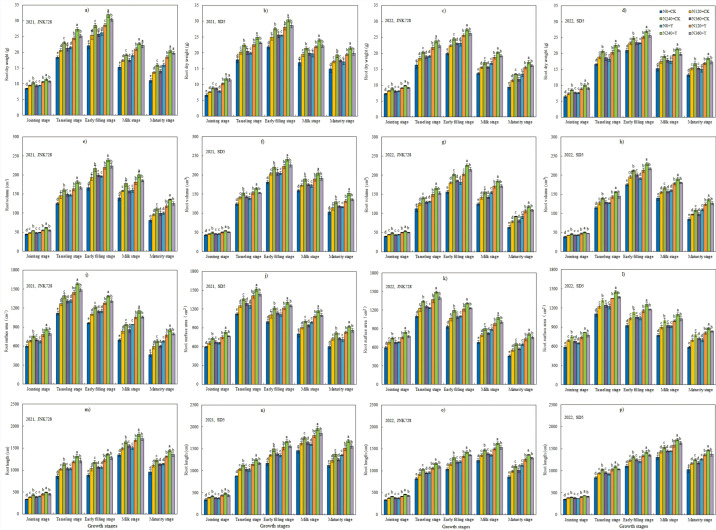
Effect of nitrogen application rates and chemical regulation on root dry weight (**a**–**d**), root surface area (**e**–**h**), root volume (**i**–**l**), and root length (**m**–**p**) in 2021 and 2022. JNK728 and SD5 indicate maize varieties Jingnongke 728 and Saide 5, respectively. N0+CK, N120+CK, N240+CK, and N360+CK indicate nitrogen application rates at 0, 120, 240, and 360 kg ha^−1^ combined with water, respectively. N0+Y, N120+Y, N240+Y, and N360+Y indicate nitrogen application rates at 0, 120, 240, and 360 kg ha^−1^ combined with plant growth regulator, respectively. Error bars indicate the value of standard error. Bars within the same growth stage marked with different letters are significantly different based on one-way ANOVA followed by Tukey’s test (*p* < 0.05).

From jointing stage to maturity stage during both 2021 and 2022, root morphological parameters (including root dry weight, root surface area, root volume, and root length) increased initially and then decreased, with maximum values occurring at either the tasseling stage or early grain-filling stage (Figure 2). Nitrogen application and chemical regulation significantly enhanced maize root growth at different growth stages under high planting density. Root dry weight, surface area, volume, and length initially increased and then decreased with increasing nitrogen application rates, reaching maximum values at the N240 treatment. Taking root dry weight at early filling stage as an example, compared with 0 kg N ha^−1^ (N0), Jingnongke (JNK728) and Saide 5 (SD5) showed root dry weight increases of 11.9%, 23.2%, and 15.5% and 10.9%, 20.3%, and 13.1% under 120 kg N ha^−1^ (N120), 240 kg N ha^−1^ (N240), and 360 kg N ha^−1^ (N360) treatments, respectively. These results indicate that moderate nitrogen application increases root dry weight, while excessive nitrogen input (N360 treatment) leads to significant reduction. Compared with the non-application of chemical regulators (CK), chemical regulation significantly increased root dry weight, root surface area, root volume, and root length by 12.8%, 12.1%, 11.6%, and 17.3%, respectively. Compared with CK, plant growth regulator (PGR) significantly increased root dry weight, root surface area, root volume, and root length by 12.8%, 12.1%, 11.6%, and 17.3%, respectively.

### 2.2. Root Bleeding Sap

#### 2.2.1. Bleeding Sap Rate

Root bleeding sap serves as a reliable indicator of root system vitality and physiological activity to a certain extent. The root bleeding sap rate exhibited a unimodal pattern during maize growth, peaking at the tasseling stage (Table 1). Nitrogen application significantly enhanced root bleeding sap rate, which reached its maximum under the N240 treatment, while further nitrogen input (N360) resulted in a significant reduction. PGR significantly enhanced root bleeding sap rate. Between the two cultivars, JNK728 exhibited higher bleeding sap rate than SD5. These results indicate that appropriate nitrogen application combined with chemical regulation significantly enhances root bleeding sap rate, improves root system vitality, and promotes soil nutrient absorption and utilization in maize under high planting density.

#### 2.2.2. Mineral Nutrient and Amino Acid Concentrations in Root Bleeding Sap

Mineral nutrient and amino acid concentrations in root bleeding sap indicated consistent decreasing trends from the jointing stage to milk stage in two years (Table 2, Table 3, Table 4 and Table 5). Nitrogen application and chemical regulation significantly affected mineral element and amino acid concentrations in root bleeding sap. With increasing nitrogen application rates, the mineral element and amino acid concentrations showed an increasing trend, reaching maximum values under the N240 treatment. PGR significantly enhanced mineral element and amino acid concentrations. These results indicate that appropriate nitrogen application combined with chemical regulation enhances root uptake capacity under high planting density, increases nutrient concentrations in root bleeding sap, and thereby provides physiological foundation for yield improvement.

### 2.3. Grain Formation

#### 2.3.1. Grain-Filling Parameters

The grain-filling process was modeled using the Logistic equation with days after anthesis as the independent variable and grain weight as the dependent variable. Grain-filling parameters for different treatments are shown in Table 6. Nitrogen application and chemical regulation significantly affected grain-filling characteristics of maize under high planting density. Nitrogen application significantly increased the maximum grain-filling rate (V_max_), mean grain-filling rate (V_m_), and active grain-filling period (P), whereas excessive nitrogen input (N360 treatment) failed to further enhance these grain-filling parameters. Chemical regulation significantly enhanced V_max_ and V_m_, showing respective increases of 5.3% and 6.2% in 2021, and 5.4% and 5.5% in 2022, compared to CK. Between the two cultivars, SD5 exhibited significantly higher V_max_ and V_m_ values than JNK728, whereas JNK728 required more days to reach maximum grain-filling rate (T_max_) compared to SD5. These results indicate that appropriate nitrogen application combined with chemical regulation significantly enhances grain-filling rate under high planting density, thereby improving the grain-filling process and ultimately promoting grain formation and grain weight increase.

#### 2.3.2. Grain Weight and Starch Content

**Figure 3:**
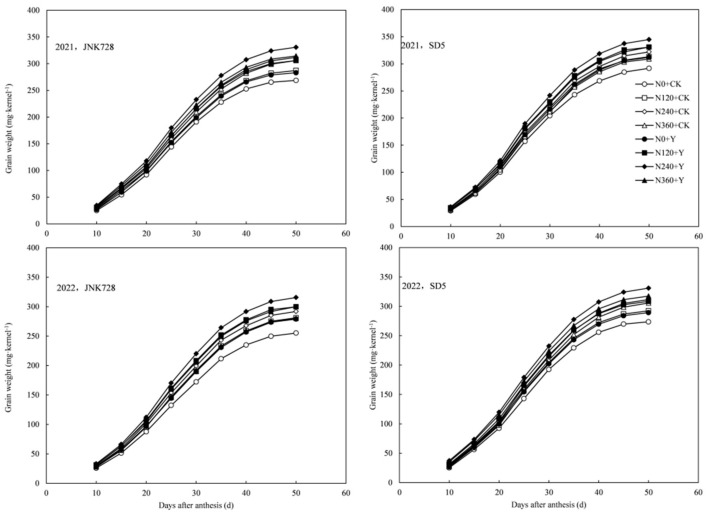
Effect of nitrogen application rates and chemical regulation on grain weight in 2021 and 2022. JNK728 and SD5 indicate maize varieties Jingnongke 728 and Saide 5, respectively. N0+CK, N120+CK, N240+CK, and N360+CK indicate nitrogen application rates at 0, 120, 240, and 360 kg ha^−1^ combined with water, respectively. N0+Y, N120+Y, N240+Y, and N360+Y indicate nitrogen application rates at 0, 120, 240, and 360 kg ha^−1^ combined with plant growth regulator, respectively.

**Figure 4:**
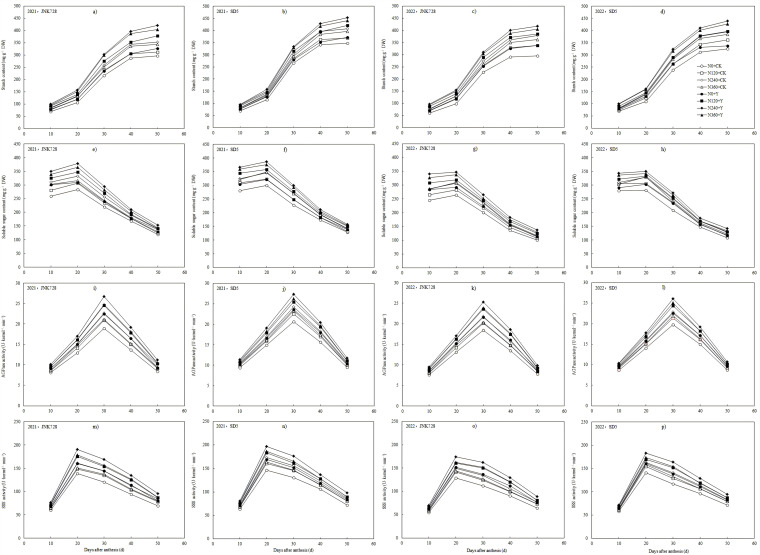
Effect of nitrogen application rates and chemical regulation on starch content (**a**–**d**), soluble sugar content (**e**–**h**), AGPase activity (**i**–**l**), and SSS activity (**m**–**p**) in grain in 2021 and 2022. JNK728 and SD5 indicate maize varieties Jingnongke 728 and Saide 5, respectively. N0+CK, N120+CK, N240+CK, and N360+CK indicate nitrogen application rates at 0, 120, 240, and 360 kg ha^−1^ combined with water, respectively. N0+Y, N120+Y, N240+Y, and N360+Y indicate nitrogen application rates at 0, 120, 240, and 360 kg ha^−1^ combined with plant growth regulator, respectively.

Grain weight and starch content exhibited sigmoidal growth patterns during the grain-filling process (Figure 3 and Figure 4a–d). Grain weight and starch accumulation increased slowly during the early filling stage, exhibited rapid growth from 20 d to 40 d after anthesis, and then gradually stabilized. Nitrogen application significantly increased grain weight and starch content during grain-filling stages, whereas excessive nitrogen input (N360) adversely affected dry matter accumulation in grains. Chemical regulation further enhanced dry matter accumulation in maize grains across all nitrogen rates. Compared with CK, PGR increased grain weight and starch content at 50 d after anthesis by 6.4% and 6.0% for JNK728, and 14.7% and 10.2% for SD5, respectively. Between the two cultivars, SD5 exhibited significantly higher grain weight and starch content than JNK728. These results indicate that appropriate nitrogen application combined with chemical regulation promotes dry matter accumulation in grains under high planting density and consequently enhances grain yield formation.

#### 2.3.3. Soluble Sugar Content in Grain

Grain soluble sugar content exhibited a unimodal pattern during grain filling, peaking at 20 days after anthesis (Figure 4e–h). Nitrogen application and chemical regulation significantly increased soluble sugar content in grains under high planting density. Grain soluble sugar content increased with elevated nitrogen application rates, whereas excessive nitrogen input (N360) significantly suppressed its accumulation. Compared with CK, PGR increased soluble sugar content by 11.9% and 10.3% in JNK728 and SD5, respectively, at 50 d anthesis. SD5 exhibited significantly higher grain soluble sugar content than JNK728 at 50 d after anthesis. These results indicate that appropriate nitrogen application combined with chemical regulation significantly enhances grain soluble sugar content under high planting density and consequently facilitates starch biosynthesis in developing grains.

#### 2.3.4. ADP-Glucose Pyrophosphorylase (AGPase) and Soluble Starch Synthase (SSS) Activities in Grain

AGPase and SSS activities in grain exhibited unimodal patterns during grain filling, peaking at 30 d and 20 d after anthesis, respectively (Figure 4i–p). AGPase and SSS activities increased with elevated nitrogen application rates, whereas excessive nitrogen input (N360) significantly suppressed both enzymatic activities. Compared with CK, PGR increased AGPase and SSS activities in JNK728 and SD5 by 10.9% and 9.6%, and 13.2% and 12.5%, respectively, at 50 d after anthesis. Between the two cultivars, SD5 exhibited significantly higher AGPase and SSS activities than JNK728. These results indicate that appropriate nitrogen application combined with chemical regulation significantly enhances AGPase and SSS activities in grain under high planting density, thereby promoting starch biosynthesis and accumulation, which ultimately improves the grain-filling process.

### 2.4. Dry Matter Accumulation

#### 2.4.1. Dry Matter Accumulation per Plant

Dry matter accumulation per plant showed rapid increase from jointing stage to milk stage, followed by gradual stabilization until reaching maximum values at maturity stage (Table 7 and Table 8). Nitrogen application significantly increased dry matter accumulation per plant, amount of dry matter per plant after anthesis (ADMA), and contribution proportion of the dry matter after anthesis (CPDMA). In 2021 and 2022, N120, N240, and N360 increased dry matter accumulation per plant at maturity stage by 15.8%, 27.0%, and 22.7% versus 11.8%, 22.0%, and 21.4%; increased ADMA by 23.2%, 38.7%, and 30.5% versus 18.1%, 31.8%, and 29.1%; and increased CPDMA by 6.3%, 9.3%, and 6.4% versus 5.6%, 8.0%, and 6.3%, respectively, compared with N0. Chemical regulation significantly reduced dry matter accumulation per plant from jointing stage to tasseling stage while markedly enhancing it from the milk stage to maturity stage. Compared with CK, PGR increased ADMA and CPDMA by 21.5% and 12.8% in 2021, and by 24.4% and 15.6% in 2022, respectively. JNK728 exhibited higher ADMA than SD5. These results indicate that nitrogen application combined with chemical regulation enhances dry matter accumulation during late growth stages under high planting density, improves post-anthesis assimilate production capacity and its contribution to grains, thereby establishing a physiological foundation for ultimate yield improvement in maize.

#### 2.4.2. Dry Matter Distribution in Different Organs at Maturity Stage

**Figure 5:**
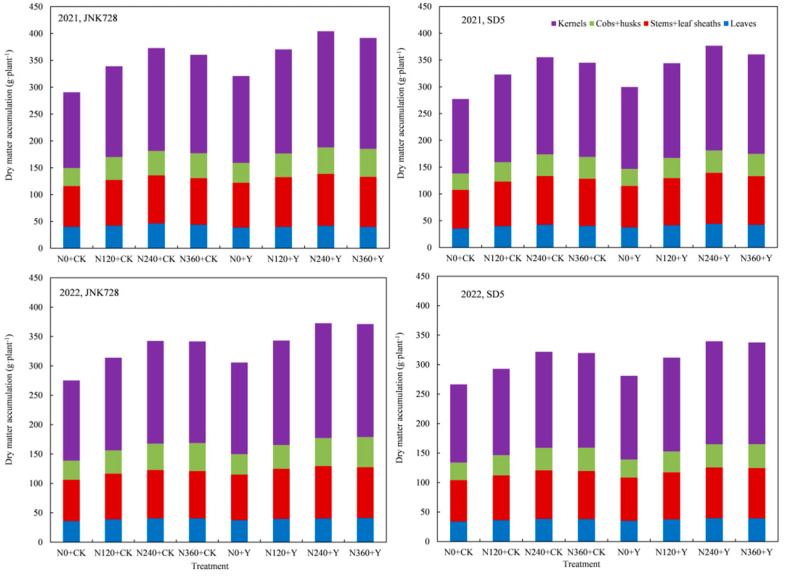
Effect of nitrogen application rates and chemical regulation on dry matter distribution in different organs at maturity stage in 2021 and 2022. JNK728 and SD5 indicate maize varieties Jingnongke 728 and Saide 5, respectively. N0+CK, N120+CK, N240+CK, and N360+CK indicate nitrogen application rates at 0, 120, 240, and 360 kg ha^−1^ combined with water, respectively. N0+Y, N120+Y, N240+Y, and N360+Y indicate nitrogen application rates at 0, 120, 240, ane 360 kg ha^−1^ combined with plant growth regulator, respectively.

Dry matter allocation (both quantity and proportion) at maturity stage among different organs followed the order: grains > stems + sheaths > leaves > cobs + husks (Figure 5). Nitrogen application significantly increased dry matter allocation across different organs. Additionally, nitrogen application reduced the dry matter allocation proportion in leaves and stems + sheaths while increasing that in cobs + husks and grains, though these differences were not significant. Compared with CK, PGR increased dry matter allocation at maturity stage by 6.7% in stems + sheaths, 5.3% in cobs + husks, and 10.4% in grains, while also enhancing the dry matter allocation proportion to grains, though this increase was not significant. These results indicate that nitrogen application combined with chemical regulation enhances both the quantity and proportion of dry matter allocated to grains at maturity stage under high planting density, thereby promoting dry matter partitioning to grains and establishing a physiological foundation for ultimate yield improvement.

### 2.5. Yield and Greenhouse Gas Emissions

#### 2.5.1. Yield

**Figure 6:**
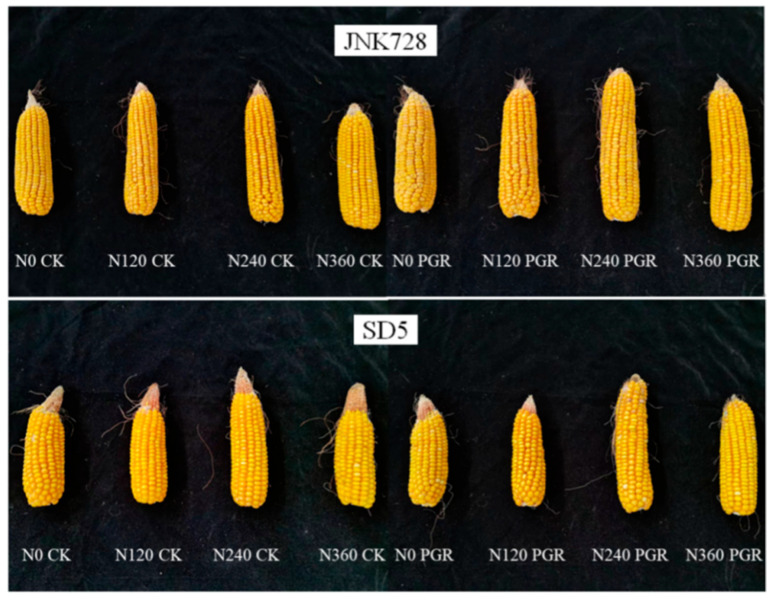
Effect of nitrogen application rates and chemical regulation on ear morphology. JNK728 and SD5 indicate maize varieties Jingnongke 728 and Saide 5, respectively. PGR and CK indicate spraying plant growth regulator and water, respectively. N0, N120, N240, and N360 indicate nitrogen application rates at 0, 120, 240, and 360 kg ha^−1^, respectively.

Nitrogen application promoted maize ear growth, increasing ear length and diameter while reducing tip barrenness, with optimal ear growth observed under the N240 treatment (Figure 6). PGR effectively reduced tip barrenness while increasing ear length and diameter, thereby significantly promoting ear growth in maize. N240 combined with PGR significantly enhanced maize yield and its components under high planting density (Table 9). N240 increased yield, grain number per ear, and 1000-grain weight by 18.4%, 9.9%, and 6.2%, respectively, compared to N0. PGR increased grain yield, grain number per ear, and 1000-grain weight by 11.4%, 9.6%, and 4.6%, respectively, compared to CK. Between the two cultivars, JNK728 exhibited significantly higher yield and grain number per ear than SD5, whereas SD5 showed greater 1000-grain weight than JNK728, though this difference was not significant.

#### 2.5.2. N_2_O and CO_2_ Cumulative Emission, Global Warming Potential (GWP), Greenhouse Gas Intensity (GHGI)

**Figure 7:**
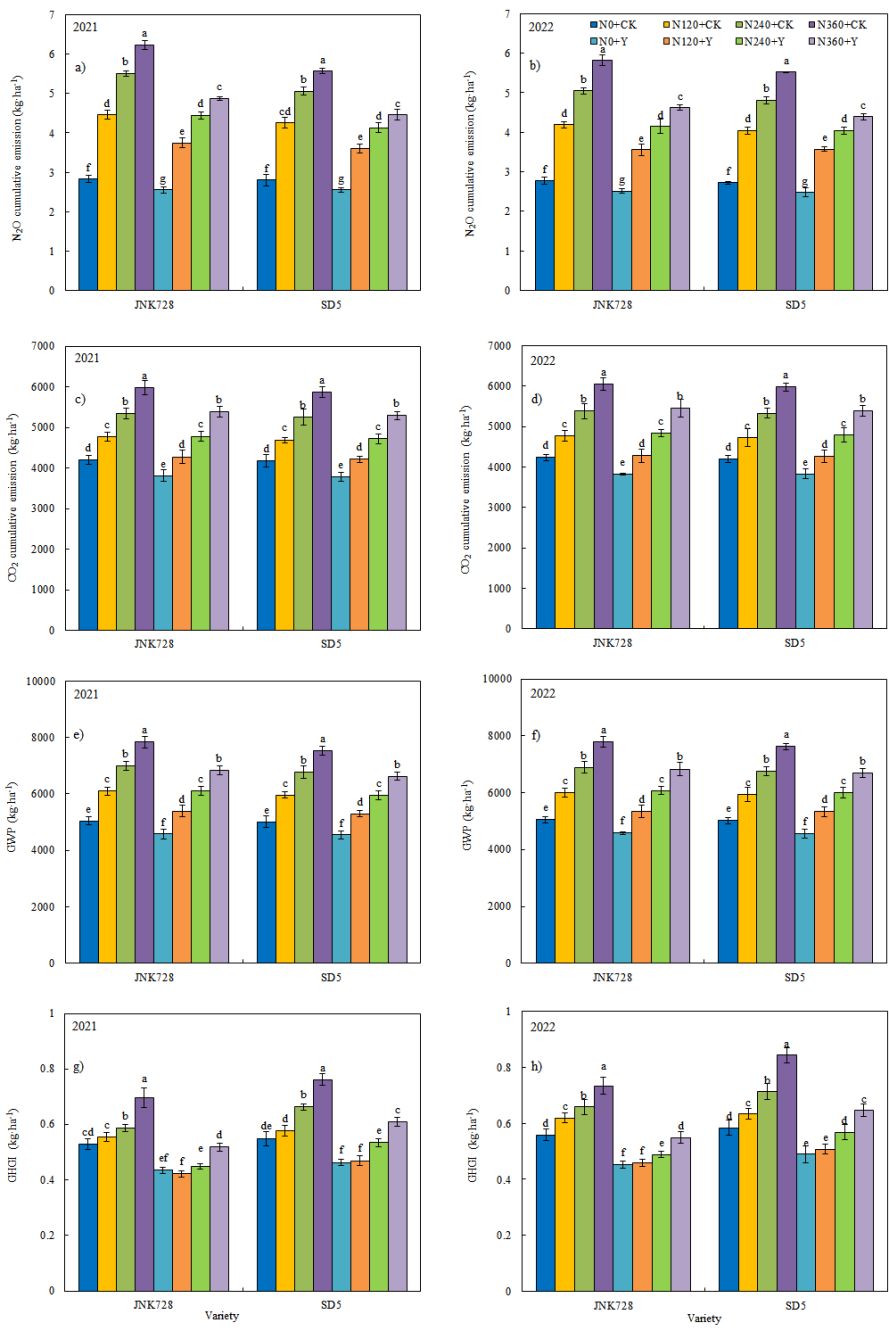
Effect of nitrogen application rates and chemical regulation on N_2_O cumulative emission (**a**,**b**), CO_2_ cumulative emission (**c**,**d**), GWP (**e**,**f**), and GHGI (**g**,**h**) in 2021 and 2022. JNK728 and SD5 indicate maize varieties Jingnongke 728 and Saide 5, respectively. N0+CK, N120+CK, N240+CK, and N360+CK indicate nitrogen application rates at 0, 120, 240, and 360 kg ha^−1^ combined with water, respectively. N0+Y, N120+Y, N240+Y, and N360+Y indicate nitrogen application rates at 0, 120, 240, and 360 kg ha^−1^ combined with plant growth regulator, respectively.

Nitrogen application significantly increased cumulative emissions of N_2_O and CO_2_, as well as GWP and GHGI, during the growing season (Figure 7). The maximum values were observed under N360, with the N_2_O cumulative emissions, CO_2_ cumulative emissions, GWP, and GHGI being 95.6%, 41.6%, 50.6%, and 31.9% higher than those under N0, respectively. Compared with CK, PGR reduced cumulative N_2_O emissions, CO_2_ emissions, GWP, and GHGI by 20.1%, 11.0%, 12.8%, and 27.4%, respectively. These findings indicate that reduced nitrogen application combined with chemical regulation under high planting density effectively mitigates greenhouse gas emissions.

## 3. Discussion

As the primary organ for nutrient uptake, roots significantly determine nutrient acquisition efficiency via their morphological and physiological traits, thereby regulating dry matter accumulation and grain yield [27,28]. High planting density frequently suppresses root growth, consequently compromising soil nutrient uptake [29]. As an effective strategy in high planting density, chemical regulation exerts a profound impact on root growth [30]. Studies have shown that under high planting density, chemical regulation can effectively optimize root size and spatial distribution, enhance soil nutrient use efficiency, and thereby improve population density tolerance to achieve high yields [31]. In this study, chemical regulation significantly increased root surface area, root volume, root length, and root dry weight. Furthermore, optimal nitrogen fertilization enhances root growth. Studies have indicated that nitrogen application facilitates root elongation in maize, increases total root length and underground biomass, enhances photosynthate allocation to roots to optimize their spatial distribution, and ultimately improves nutrient uptake capacity, thereby promoting shoot growth [32,33]. In this study, nitrogen fertilizer significantly enhanced root growth under high planting density, with distinct effects observed across different application rates. Specifically, the N240 nitrogen treatment exerted the optimal promotional effect. However, excessive nitrogen application inhibited root growth, which is consistent with previous findings [8,34]. These indicate that appropriate nitrogen application promotes root growth under high planting density, whereas excessive nitrogen application impairs root growth, reduces nutrient uptake capacity, and thereby decreases dry matter accumulation in both root and aboveground plants. This phenomenon partially accounts for the yield plateau observed under high nitrogen input rates [8]. Therefore, this study confirms that optimized nitrogen management combined with chemical regulation effectively alleviates the adverse effects of high planting density on maize roots, enhances root growth and nutrient acquisition, and ultimately improves plant performance and yield formation.

Additionally, the nutrient composition of root bleeding sap can reflect the nutritional status of plants and root nutrient uptake capacity [35]. Specifically, mineral elements and amino acids in bleeding sap act as key mediators in root–shoot communication, exerting significant impacts on plant growth and nutrient utilization [36]. In this study, nitrogen application and chemical regulation increased mineral elements and amino acids concentrations in root bleeding sap, with maximum values observed under N240 and chemical regulation. These results indicate that appropriate nitrogen management combined with chemical regulation enhances root nutrient uptake and assimilation, promotes plant growth, and thereby establishes a physiological foundation for maize yield.

Dry matter accumulation and translocation serve as fundamental determinants of maize yield, providing the essential material basis for grain formation [32,37]. In this study, the combination of N240 and chemical regulation significantly increased dry matter accumulation per plant, ADMA and CPDMA, thereby laying a physiological foundation for grain growth and yield formation. It is generally recognized that photosynthates produced during the late growth stage constitute the primary source for yield formation [38]. Increasing dry matter accumulation and enhancing its allocation to grains constitute a crucial strategy for achieving high yields [39]. Our results indicated that nitrogen fertilizer and chemical regulation increased dry matter partitioning to developing grains. This result may be attributed to the enhanced leaf photosynthetic capacity after anthesis and prolonged photosynthetic duration under high planting density, as mediated by nitrogen fertilizer and chemical regulation. These effects collectively enhanced photosynthate translocation to grains, ultimately providing the physiological basis for yield improvement.

Grain growth status significantly affects final grain weight in maize [40]. Optimizing grain growth and increasing grain weight through appropriate management practices constitute a critical strategy for high-yield maize cultivation [41]. In this study, the combined application of N240 and chemical regulation significantly enhanced grain weight, indicating that these treatments improved assimilate supply to developing grains, thereby promoting grain growth and grain weight. Grain filling constitutes a complex physiological process encompassing the translocation and deposition of photoassimilates, root-absorbed nutrients, and remobilized reserves into developing grains [42]. Grain-filling characteristics, particularly filling rate and duration, are key determinants of final grain weight [43]. Studies have shown that variations in grain weight arise from altered assimilate competition within plants, leading to differences in grain-filling rate and duration [44]. The grain-filling process is strongly influenced by environmental factors, while various agronomic practices (including planting density, nitrogen application, and chemical regulation) significantly affect grain-filling dynamics [19,45,46]. Our results demonstrated that nitrogen fertilizer and chemical regulation significantly increased the maximum grain-filling rate, mean grain-filling rate, and active grain-filling period. This enhancement may be attributed to improved plant productivity and increased dry matter accumulation, which meet grain demand and optimize the grain-filling process.

Starch represents the predominant storage compound in maize grains, comprising approximately 70% of grain weight [47]. Accordingly, the grain-filling process in maize is primarily characterized by starch biosynthesis and accumulation in developing grains. AGPase and SSS are key enzymes catalyzing photosynthates’ conversion into total starch and amylopectin, ultimately determining the final starch content and composition of grains [48,49]. In this study, nitrogen application and chemical regulation enhanced AGPase and SSS activities in grains. This facilitated starch biosynthesis and accumulation, thereby optimizing the grain-filling process, which is consistent with previous studies [50]. During grain filling, translocated photosynthates initially exist as soluble sugars in grains prior to being converted to starch [51]. Therefore, the soluble sugar content in grains is closely correlated with starch accumulation and can reflect the potential for starch synthesis [52]. Our results indicated a unimodal pattern in the soluble sugar content during grain filling, characterized by an initial increase followed by a decrease. This pattern reflects the following: (1) the translocation of assimilates from vegetative organs (leaves, sheaths, stems) to grains during the early grain-filling stage, which results in increased soluble sugar levels; and (2) subsequently soluble sugar is converted to starch as grain-filling progresses, leading to a reduction in soluble sugar content [53]. In this study, nitrogen application and chemical regulation increased soluble sugar content in grains during grain filling, consistent with previous study [54]. It may be attributed to enhanced metabolic activity in leaves, roots, and stems through nitrogen and chemical regulation, thereby improving photosynthate translocation to grains during the filling period [55]. Furthermore, nitrogen fertilizer sustained active carbon metabolism in developing grains, ensuring adequate photoassimilate supply for grain growth requirements [54]. Chemical regulation promoted soluble sugar metabolism in leaves and grains, facilitating assimilate transport to grains [46]. Insufficient soluble sugar can limit starch biosynthesis in grains [56]. However, nitrogen fertilizer and chemical regulation sustained elevated levels of photosynthetic assimilates in grains, thereby sufficiently meeting the growth requirements of grains.

Increasing nitrogen application constitutes a critical strategy for achieving high yields, which has contributed to a rising trend in nitrogen input in recent years. In this study, nitrogen fertilizer and chemical regulation significantly increased grain number per ear, 1000-grain weight, and yield under high planting density. However, once the nitrogen application rate exceeds optimal levels, its positive effects on maize yield and yield component diminished, and it potentially even showed declining trends [57]. Furthermore, our results indicated that elevated nitrogen application significantly increased N_2_O and CO_2_ emissions. It may associate with changes in the expression abundance of microorganisms associated with the nitrogen cycle [58]. Notably, N_2_O serves as the primary form of gaseous nitrogen loss from fertilizers; its emissions reduce nitrogen use efficiency and cause environmental risks [59]. It has been demonstrated that excessive nitrogen fertilizer input fails to promote yield enhancement and induces resource waste and environmental pollution [60,61]. Studies have shown that increasing yield is a crucial approach for improving nitrogen use efficiency. Therefore, to achieve both high yield and efficient nitrogen utilization, it is necessary to explore yield potential rather than overly rely on high nitrogen inputs. In this study, chemical regulation reduced N_2_O and CO_2_ emissions while maximizing yield. It may be achieved by enhancing nitrogen utilization efficiency, thereby realizing high-yield and high-efficiency crop production [62,63]. Efficient nitrogen utilization directly contributes to reduced N_2_O emissions. Dry matter synthesis and yield increase will enhance nitrogen uptake, thereby reducing soil N_2_O emissions, as well as GWP and GHGI [64]. In this study, the combination of N240 and chemical regulation optimally balanced yield potential with nitrogen loss, demonstrating significant importance for improving maize production and alleviating environmental pressure.

## 4. Materials and Methods

### 4.1. Field Sites

This study was carried out at the Minzhu experimental station of Harbin Academy of Agricultural Sciences (45°75′ N, 126°63′ E; 140 m a.s.l.) in Heilongjiang Province during 2021–2022. The site is characterized by a temperate continental monsoon climate, with annual averages of 569.1 mm precipitation (65% in summer), 4.3 °C temperature, 2642.1 h sunshine duration, 1324.3 mm evaporation, and a 140–150-day frost-free period. Meteorological data during the maize growing seasons are obtained from the Harbin Academy of Agricultural Sciences and provided in Table 10. The experiment was conducted on typical chernozem under a continuous maize cropping system. The 0–20 cm soil layer exhibited the following properties: pH 6.75; organic matter 1.773 g kg^−1^; total nitrogen 0.513 g kg^−1^; total phosphorus 0.252 g kg^−1^; total potassium 2.933 g kg^−1^; available nitrogen 220.38 mg kg^−1^; available phosphorus 59.85 mg kg^−1^; and available potassium 131.24 mg kg^−1^. Soil analysis methods were performed according to reference [65].

### 4.2. Experimental Design and Field Management

The experiment was conducted using a split-split plot design involving three factors: cultivar as the main plot, chemical regulation as the subplot, and nitrogen application as the sub-subplot. Two maize cultivars, namely JNK728 and SD5, were assigned to the main plots, with two chemical regulation treatments allocated to the subplots and four nitrogen rates to the sub-subplots. Compared with JNK728, SD5 exhibits more compact plant figure with smaller individual plant coverage area. The plant growth regulator used was 30% diethyl aminoethyl hexanoate·ethephon (containing 3% DA-6 and 27% ethephon), commercially named Guoguang Aifeng (Sichuan Runer Technology Co., Ltd., Chengdu, China). For each cultivar treatment, two chemical regulation treatments were established: PGR treatment involved uniform foliar application of 0.83 mL L^−1^ regulator solution at the seven-leaf stage using a spray volume of 450 L ha^−1^, while CK treatment received an equivalent volume of water. Four nitrogen rates were assigned under each chemical regulation treatment: 0 (N0), 120 (N120), 240 (N240), and 360 (N360) kg N ha^−1^, totaling sixteen treatments with three replicates. Seeds were manually sown in late April each year at approximately 5 cm depth, with a planting density of 90,000 plants ha^−1^. Each plot consisted of 10 rows with 65 cm row spacing and 8 m row length. Nitrogen fertilizer (urea) was applied as 50% basal dressing and 50% topdressing at the jointing stage, while phosphorus (100 kg P_2_O_5_ ha^−1^) and potassium (100 kg K_2_O ha^−1^) fertilizers were applied entirely as basal dressing. All plots were harvested on September 25 annually. No irrigation was applied during the maize growing season. Pests, weeds, and diseases were controlled in a timely manner, and tillage management was conducted according to local farmer management.

### 4.3. Sampling and Measurements

#### 4.3.1. Root Morphology and Root Bleeding Sap

Root sampling was conducted at jointing, tasseling, early filling, milk, and maturity stages, with three consecutive plants excavated during each sampling event. The root system was sampled using the profile method within a 20 cm radius from the plant base (0–40 cm soil depth) [36]. After washing with clean water, roots were scanned using an Epson V700 root scanner (Epson Co., Ltd., Jakarta, Indonesia) and analyzed with WinRHIZO version 5.0 (Regent Instruments Inc., Quebec City, QC, Canada) to determine root length, surface area, and volume. The scanned roots were oven-dried at 80 °C to constant weight for dry weight measurement [36]. Root bleeding sap was collected from 19:00 to 07:00 the next day at each growth stage. A suitable amount of dry absorbent cotton (approximately 2/3 of the tube volume) was placed into test tubes. The stems were then rapidly cut at the third basal internode using scissors, and the tubes were secured to the residual stumps with plastic film for bleeding sap collection. The bleeding rate, mineral elements concentrations, and amino acids concentrations in root bleeding sap were determined following Sun et al. [66].

#### 4.3.2. Grain Filling, Grain Formation and Yield

At the silking stage, five uniform plants were selected. From 10 d to 50 d after anthesis, samples were collected at 5-day intervals. For each sampling, 100 middle-positioned grains were excised from ears, inactivated at 105 °C for 30 min, and then dried at 80 °C to determine grain dry weight. The grain-filling process was fitted using the Logistic equation and parameters were calculated according to Ren et al. [67]. At 10 d, 20 d, 30 d, 40 d, and 50 d after anthesis, three ears per treatment were collected, and middle-positioned grains were sampled for determination. Soluble sugar and starch content were measured following Wang et al. [68], and AGPase and SSS activities were measured following Zhang et al.’s method [69]. At maturity stage, the central three rows of maize plants per plot were harvested for determining yield (adjusted to 14% moisture content), grain number per ear, and 1000-grain weight measurement.

#### 4.3.3. Dry Matter Accumulation

Plants were sampled at the jointing, tasseling, early filling, milking, and maturity stages, with three representative plants of uniform growth selected per treatment. The plants were separated into different organs (leaves, stems + sheaths, cob + husks, and grains), which were then inactivated at 105 °C for 30 min and oven-dried at 80 °C to constant weight for dry weight measurement. ADMA and CPDMA were calculated according to Gao et al. [70]:

ADMA = dry matter accumulation per plant at maturity stage − dry matter accumulation per plant at anthesis;

CPDMA = post-anthesis dry matter accumulation per plant/dry matter accumulation per plant at maturity stage.

#### 4.3.4. Greenhouse Gas Emissions

As this study was conducted under rainfed conditions, only N_2_O and CO_2_ emissions were considered, while CH_4_ emissions were excluded. N_2_O and CO_2_ emissions were measured throughout the maize growing season using the static chamber-gas chromatography method according to Dyer et al. [71]. The sampling chamber was constructed of opaque PVC material with dimensions of 60 cm (length) × 25 cm (width) × 30 cm (height). The chamber top was equipped with a three-way valve for gas sampling and contained a small fan to ensure homogeneous gas mixing prior to sampling. The chamber base was inserted 10 cm into the soil, with the chamber body securely fitted into the groove of the base. The groove was water-sealed to ensure airtight conditions. During measurement periods, the chambers remained free of crops and weeds and were positioned between crop rows within each plot. During the entire maize growing season, gas sampling was conducted at 7-day intervals. Additional sampling was performed 1 day after topdressing, followed by resumption of the 7-day interval after one week. All sampling occurred between 09:00 and 11:00 h. Following chamber closure, gas samples were collected by opening the stopcock valve at 0, 15, and 30 min, and withdrawing gas with a syringe. Collected samples were immediately sealed and analyzed on the same day. The concentrations of N_2_O and CO_2_ were determined using a gas chromatograph (Agilent 7890B, Agilent Technologies, Inc., Shanghai, China), with calibration and quantification performed using high-purity standards (0.5 mg L^−1^). The cumulative emissions of N_2_O and CO_2_ (kg ha^−1^) were calculated by linear interpolation between successive sampling dates [72].

GWP was calculated as follows: GWP is used to represent the cumulative radiative forcing of a unit mass of greenhouse gas over a specific time horizon. In the calculation, CO_2_ was used as the reference gas (GWP = 1 for CO_2_). For a 100-year time horizon of climate change, the GWP of N_2_O is 298 times that of CO_2_. The calculation formula followed Yang et al. [73]: GWP (kg ha^−1^) = CO_2_ cumulative emissions (kg ha^−1^) + N_2_O cumulative emissions (kg ha^−1^) × 298.

GHGI was calculated as follows: GHGI is defined as the greenhouse gas emissions per unit of economic output. The calculation formula followed Yang et al. [73]: GHGI = GWP/Y, where Y represents yield (kg ha^−1^).

### 4.4. Data Analysis

Statistical analyses were performed using SPSS Statistics 21.0 (SPSS Inc., Chicago, IL, USA) with two-way analysis of variance (ANOVA) and least significant difference (LSD) test at *p* < 0.05 to determine treatment effects of nitrogen fertilizer and chemical regulation on the following: (i) root morphological traits, (ii) root bleeding rate, (iii) grain formation, (iv) dry matter accumulation, (v) field greenhouse gas emissions, and (vi) yield parameters. The grain-filling dynamics were fitted using Curve Expert 1.3 software. Figures were prepared using Microsoft Excel 2010.

## 5. Conclusions

**Figure 8:**
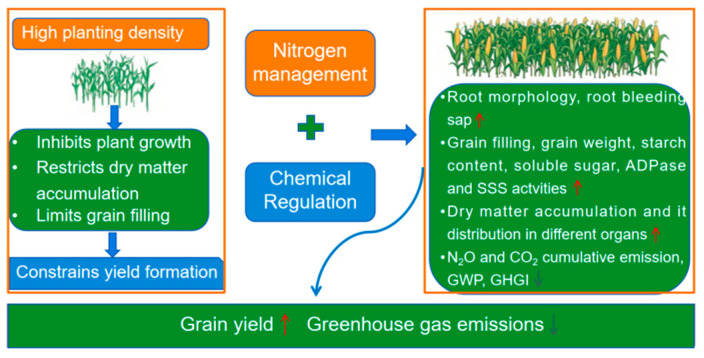
Effects of nitrogen fertilizer and chemical regulation on root growth, grain filling, dry matter accumulation and greenhouse gas emissions. The red upward arrow represents an increase in its content, and the gray downward arrow represents a decrease in its content.

In this study, nitrogen fertilization and chemical regulation exert a positive effect on maize yield formation. Under high planting density, the combination of 240 kg N ha^−1^ and chemical regulation enhances root growth, optimizes dry matter allocating to grains, reduces greenhouse gas emissions, and increases maize yield (Figure 8). Consequently, this cultivation practice provides a theoretical basis for high-yield and high-efficiency maize production under high planting density.

## Figures and Tables

**Table 1:** Effect of nitrogen application rates and chemical regulation on root bleeding sap rate (g plant^−1^ h^−1^).

Treatment	2021					2022				
	Jointing Stage	Tasseling Stage	Early Filling Stage	Milk Stage	Maturity Stage	Jointing Stage	Tasseling Stage	Early Filling Stage	Milk Stage	Maturity Stage
Nitrogen application										
N0	2.48 c	2.55 c	2.22 c	1.96 c	0.92 c	2.19 c	2.23 c	1.97 c	1.70 c	0.72 c
N120	2.79 b	2.98 b	2.63 b	2.23 b	1.08 b	2.47 b	2.64 b	2.28 b	1.99 b	0.80 b
N240	3.07 a	3.39 a	2.98 a	2.45 a	1.21 a	2.81 a	3.05 a	2.54 a	2.23 a	0.89 a
N360	2.85 b	3.07 b	2.69 b	2.27 b	1.11 b	2.53 b	2.77 b	2.35 b	2.04 b	0.82 b
Chemical regulation										
CK	2.66 b	2.83 b	2.52 b	2.21 b	1.01 b	2.33 b	2.51 b	2.07 b	1.66 b	0.73 b
PGR	2.94 a	3.17 a	2.74 a	2.34 a	1.15 a	2.67 a	2.83 a	2.35 a	1.92 a	0.89 a
Variety										
JNK728	2.89 a	3.08 a	2.70 a	2.30 a	1.11 a	2.63 a	2.77 a	2.29 a	1.86 a	0.84 a
SD5	2.71 b	2.92 b	2.56 b	2.16 b	1.05 b	2.37 b	2.57 b	2.13 b	1.72 b	0.78 b
Sources of variation										
V	**	*	*	**	**	**	**	**	**	**
N	**	**	**	**	**	**	**	**	**	**
C	**	**	**	**	**	**	**	**	**	**
V × N	NS	*	*	*	NS	NS	*	*	*	NS
V × C	*	NS	*	*	*	*	*	NS	*	*
N × C	NS	NS	NS	NS	NS	NS	NS	NS	NS	NS
V × N × C	NS	NS	NS	NS	NS	NS	NS	NS	NS	NS

N0, N120, N240, and N360 indicate nitrogen application rates at 0, 120, 240, and 360 kg ha^−1^, respectively. PGR and CK indicate spraying plant growth regulator and water, respectively. JNK728 and SD5 indicate maize varieties Jingnongke 728 and Saide 5, respectively. V, N, and C indicate variety, nitrogen application, and chemical regulation, respectively. Values within a column for the same treatment followed by the different letters indicate a significant difference at *p* < 0.05. * and ** indicate significance at the 0.05 and 0.01 probability levels, respectively, and NS is not significant.

**Table 2:** Effect of nitrogen application rates and chemical regulation on mineral nutrient concentrations in root bleeding sap (μg plant^−1^ h^−1^) (2021).

Treatment	Mineral Elements									
	Fe	Mn	Cu	Zn	Ca	Mg	K	P	B	Si
Jointing stage										
Nitrogen application										
N0	1.16 c	4.62 c	0.348 c	11.27 c	321.7 c	302.2 c	1788 c	116.6 c	1.19 c	49.91 c
N120	1.31 b	5.04 b	0.382 b	12.14 b	348.4 b	320.1 b	1947 b	128.3 b	1.32 b	53.53 b
N240	1.54 a	5.46 a	0.419 a	12.91 a	376.9 a	339.3 a	2109 a	139.7 a	1.44 a	57.32 a
N360	1.47 a	5.40 a	0.410 a	12.73 a	368.4 a	322.4 b	2022 a	133.3 a	1.33 b	56.03 a
Chemical regulation										
CK	1.25 b	4.60 b	0.367 b	11.60 b	334.8 b	302.5 b	1856 b	119.3 b	1.24 b	51.05 b
PGR	1.49 a	5.67 a	0.412 a	12.93 a	372.9 a	339.4 a	2077 a	139.6 a	1.40 a	57.34 a
Variety										
JNK728	1.41 a	5.29 a	0.399 a	12.66 a	363.9 a	329.6 a	2029 a	133.3 a	1.36 a	55.59 a
SD5	1.33 b	4.98 b	0.380 b	11.87 b	343.8 b	312.4 b	1904 b	125.6 b	1.28 b	52.80 b
Sources of variation										
V	**	**	*	**	**	**	**	**	**	*
N	**	**	**	**	**	**	**	**	**	**
C	**	**	**	**	**	**	**	**	**	**
V × N	NS	*	NS	NS	*	NS	NS	*	*	NS
V × C	NS	NS	*	*	NS	*	**	*	NS	NS
N × C	NS	NS	NS	NS	NS	NS	NS	NS	NS	NS
V × N × C	NS	NS	NS	NS	NS	NS	NS	NS	NS	NS
Tasseling stage										
Nitrogen application										
N0	0.627 c	4.43 c	0.502 c	9.79 c	308.6 c	291.8 c	1608 c	105.4 c	1.08 c	45.83 c
N120	0.755 b	4.86 b	0.545 b	10.57 b	332.17 b	312.7 b	1727 b	118.0 b	1.23 b	49.19 b
N240	0.880 a	5.32 a	0.595 a	11.32 a	351.46 a	330.4 a	1847 a	130.8 a	1.35 a	52.72 a
N360	0.875 a	5.32 a	0.590 a	11.33 a	330.75 b	320.0 ab	1805 ab	122.0 b	1.26 b	52.81 a
Chemical regulation										
CK	0.725 b	4.71 b	0.519 b	10.02 b	308.9 b	299.1 b	1647 b	110.9 b	1.15 b	46.88 b
PGR	0.844 a	5.25 a	0.597 a	11.48 a	352.6 a	328.3 a	1846 a	127.2 a	1.30 a	53.39 a
Variety										
JNK728	0.805 a	5.14 a	0.573 a	11.04 a	340.4 a	322.3 a	1795 a	122.3 a	1.26 a	51.75 a
SD5	0.764 b	4.82 b	0.543 b	10.46 b	321.1 b	305.2 b	1698 b	115.7 b	1.20 b	48.52 b
Sources of variation										
V	*	**	*	*	**	*	*	*	*	**
N	**	**	**	*	*	*	*	**	**	**
C	**	**	**	**	**	**	**	**	**	**
V × N	*	*	*	NS	*	*	NS	*	*	NS
V × C	NS	*	NS	*	NS	NS	*	NS	NS	*
N × C	NS	NS	NS	NS	NS	*	NS	NS	NS	NS
V × N × C	NS	NS	NS	NS	NS	NS	NS	NS	NS	NS
Milk stage										
Nitrogen application										
N0	0.501 c	1.66 c	0.149 c	3.94 c	130.5 c	31.37 d	534.1 d	47.9 c	0.275 c	18.25 c
N120	0.575 b	1.89 b	0.163 b	4.34 b	147.0 b	37.76 c	612.8 c	54.8 b	0.310 b	20.74 b
N240	0.660 a	2.27 a	0.181 a	4.69 a	163.3 a	47.14 a	698.7 a	61.2 a	0.335 a	23.47 a
N360	0.655 a	2.21 a	0.172 ab	4.73 a	157.5 a	44.63 b	652.0 b	56.5 b	0.285 c	23.34 a
Chemical regulation										
CK	0.528 b	1.77 b	0.149 b	4.13 b	140.7 b	35.03 b	580.5 b	51.7 b	0.272 b	19.44 b
PGR	0.668 a	2.24 a	0.183 a	4.71 a	158.5 a	45.42 a	668.4 a	58.5 a	0.331 a	23.46 a
Variety										
JNK728	0.613 a	2.07 a	0. 171 a	4.57 a	153.3 a	41.33 a	640.6 a	56.7 a	0.309 a	22.17 a
SD5	0.583 b	1.94 b	0. 162 b	4.28 b	145.8 b	39.12 b	608.3 b	53.4 b	0.293 b	20.73 b
Sources of variation										
V	*	**	*	**	*	*	*	**	*	**
N	**	**	**	**	**	**	**	**	**	**
C	**	**	**	**	**	**	**	**	**	**
V × N	*	*	*	NS	NS	*	*	*	NS	*
V × C	NS	NS	NS	NS	*	*	*	*	NS	NS
N × C	NS	NS	*	NS	NS	NS	*	NS	NS	NS
V × N × C	NS	NS	NS	NS	NS	NS	*	NS	NS	NS

N0, N120, N240, and N360 indicate nitrogen application rates at 0, 120, 240, and 360 kg ha^−1^, respectively. PGR and CK indicate spraying plant growth regulator and water, respectively. JNK728 and SD5 indicate maize varieties Jingnongke 728 and Saide 5, respectively. V, N, and C indicate variety, nitrogen application, and chemical regulation, respectively. Values within a column for the same treatment followed by the different letters indicate a significant difference at *p* < 0.05. * and ** indicate significance at the 0.05 and 0.01 probability levels, respectively, and NS is not significant.

**Table 3:** Effect of nitrogen application rates and chemical regulation on mineral nutrients concentrations in root bleeding sap (μg plant^−1^ h^−1^) (2022).

Treatment	Mineral Elements									
	Fe	Mn	Cu	Zn	Ca	Mg	K	P	B	Si
Jointing stage										
Nitrogen application										
N0	1.45 c	3.75 c	0.291 c	9.54 c	284.5 c	256.5 c	1564 c	97.5 c	0.984 c	43.77 c
N120	1.68 b	4.16 b	0.322 b	10.51 b	310.6 b	279.4 b	1715 b	108.9 b	1.075 b	47.41 b
N240	1.82 a	4.60 a	0.355 a	11.53 a	340.2 a	302.7 a	1861 a	119.4 a	1.160 a	51.14 a
N360	1.79 a	4.68 a	0.345 a	11.24 a	329.7 a	281.6 b	1794 ab	114.6 a	1.055 b	49.93 ab
Chemical regulation										
CK	1.56 b	3.82 b	0.310 b	10.01 b	295.5 b	261.9 b	1621 b	102.3 b	0.995 b	44.66 b
PGR	1.81 a	4.77 a	0.346 a	11.39 a	337.0 a	298.2 a	1846 a	118.0 a	1.142 a	51.46 a
Variety										
JNK728	1.74 a	4.41 a	0.338 a	10.97 a	324.7 a	289.3 a	1780 a	113.6 a	1.095 a	49.64 a
SD5	1.63 b	4.18 b	0.318 b	10.43 b	307.8 b	270.8 b	1687 b	106.7 b	1.042 b	46.48 b
Sources of variation										
V	**	*	**	*	**	**	**	**	*	**
N	**	**	**	**	**	**	**	**	**	**
C	**	**	**	**	**	**	**	**	**	**
V × N	NS	*	*	NS	*	NS	NS	*	*	NS
V × C	*	*	NS	*	NS	*	*	*	NS	*
N × C	NS	NS	NS	NS	NS	NS	NS	NS	NS	NS
V × N × C	NS	NS	NS	NS	NS	NS	NS	NS	NS	NS
Tasseling stage										
Nitrogen application										
N0	0.413 c	3.75 c	0.399 c	8.42 c	274.5 c	260.5 c	1399 c	96.5 c	1.032 c	40.88 c
N120	0.505 b	4.17 b	0.441 b	9.12 b	291.7 b	280.0 b	1517 b	104.6 b	1.045 b	43.54 b
N240	0.646 a	4.63 a	0.490 a	9.75 a	311.9 a	297.8 a	1637 a	113.9 a	1.140 a	46.18 a
N360	0.634 a	4.64 a	0.485 a	9.66 a	297.5 ab	286.9 ab	1594 a	106.7 b	1.075 b	45.25 ab
Chemical regulation										
CK	0.513 b	4.01 b	0.415 b	8.65 b	278.2 b	267.3 b	1446 b	99.6 b	1.002 b	41.85 b
PGR	0.586 a	4.59 a	0.492 a	9.82 a	309.6 a	295.4 a	1628 a	111.3 a	1.144 a	46.07 a
Variety										
JNK728	0.564 a	4.41 a	0.466 a	9.51 a	302.4 a	289.3 a	1578 a	108.3 a	1.105 a	45.29 a
SD5	0.535 b	4.18 b	0.441 b	8.96 b	285.4 b	273.4 b	1496 b	102.6 b	1.041 b	42.63 b
Sources of variation										
V	**	*	**	*	**	**	**	**	**	**
N	**	**	**	**	**	**	**	**	**	*
C	**	**	**	**	**	**	**	**	**	**
V × N	*	*	NS	*	*	*	NS	*	*	NS
V × C	NS	*	*	NS	NS	*	*	*	NS	*
N × C	NS	NS	*	NS	NS	NS	NS	NS	NS	NS
V × N × C	NS	NS	NS	NS	NS	NS	NS	NS	NS	NS
Milk stage										
Nitrogen application										
N0	0.331 c	1.07 c	0.110 d	2.64 c	103.4 c	16.32 c	425.6 c	36.39 c	0.181 d	13.34 c
N120	0.390 b	1.29 b	0.126 c	3.03 b	115.4 b	22.24 b	487.2 b	41.94 b	0.213 c	14.83 b
N240	0.445 a	1.64 a	0.143 a	3.58 a	128.8 a	30.10 a	552.9 a	47.73 a	0.245 a	16.21 a
N360	0.405 b	1.69 a	0.134 b	3.56 a	124.2 a	28.76 a	527.7 a	45.60 a	0.230 b	15.73 a
Chemical regulation										
CK	0.350 b	1.26 b	0.118 b	2.97 b	110.4 b	21.13 b	463.8 b	40.50 b	0.198 b	13.34 b
PGR	0.436 a	1.58 a	0.138 a	3.43 a	125.5 a	27.58 a	532.9 a	45.33 a	0.236 a	16.71 a
Variety										
JNK728	0.405 a	1.47 a	0.132 a	3.29 a	121.5 a	25.06 a	511.8 a	44.05 a	0.225 a	15.42 a
SD5	0.381 b	1.37 b	0.124 b	3.11 b	114.4 b	23.65 b	484.8 b	41.78 b	0.210 b	14.63 b
Sources of variation										
V	**	**	**	**	**	**	**	*	**	*
N	**	**	**	**	**	**	**	**	**	**
C	**	**	**	**	**	**	**	**	**	**
V × N	*	*	*	NS	NS	*	*	*	NS	*
V × C	NS	*	NS	*	*	*	NS	*	*	NS
N × C	NS	NS	NS	NS	NS	NS	*	NS	NS	NS
V × N × C	NS	NS	NS	NS	NS	NS	NS	NS	NS	NS

N0, N120, N240, and N360 indicate nitrogen application rates at 0, 120, 240, and 360 kg ha^−1^, respectively. PGR and CK indicate spraying plant growth regulator and water, respectively. JNK728 and SD5 indicate maize varieties Jingnongke 728 and Saide 5, respectively. V, N, and C indicate variety, nitrogen application, and chemical regulation, respectively. Values within a column for the same treatment followed by the different letters indicate a significant difference at *p* < 0.05. * and ** indicate significance at the 0.05 and 0.01 probability levels, respectively, and NS is not significant.

**Table 4:** Effect of nitrogen application rates and chemical regulation on amino acids concentrations in root bleeding sap (μg plant^−1^ h^−1^) (2021).

Treatment	Ser	Glu	Gly	Ala	Val	Lys	Met	Arg	Leu
Jointing stage									
Nitrogen application									
N0	475.0 c	293.6 c	1.34 c	14.37 c	57.78 c	97.57 c	5.40 c	86.09 c	17.11 c
N120	514.5 b	319.6 b	1.46 b	15.41 b	63.60 b	103.36 b	5.82 b	93.99 b	19.36 b
N240	556.6 a	346.3 a	1.59 a	16.50 a	69.24 a	109.58 a	6.19 a	101.88 a	22.65 a
N360	544.1 a	332.0 ab	1.58 a	16.13 ab	66.06 ab	104.10 b	6.10 ab	100.68 a	21.71 a
Chemical regulation									
CK	496.2 b	298.8 b	1.39 b	14.66 b	60.72 b	97.88 b	5.53 b	90.45 b	18.92 b
PGR	548.9 a	347.0 a	1.60 a	16.54 a	67.62 a	109.42 a	6.23 a	100.87 a	21.49 a
Variety									
JNK728	543.9 a	334.4 a	1.54 a	16.09 a	66.29 a	107.55 a	6.06 a	99.29 a	20.91 a
SD5	501.2 b	311.4 b	1.45 b	15.11 b	62.05 b	99.75 b	5.70 b	92.03 b	19.50 b
Sources of variation									
V	**	**	**	**	**	**	**	**	**
N	**	**	**	**	**	*	**	**	**
C	**	**	**	**	**	**	**	**	**
V × N	NS	NS	NS	NS	NS	NS	NS	NS	NS
V × C	NS	NS	NS	NS	NS	*	NS	NS	NS
N × C	NS	*	NS	NS	NS	NS	NS	NS	NS
V × N × C	NS	NS	NS	NS	NS	NS	NS	NS	NS
Tasseling stage									
Nitrogen application									
N0	368.8 c	232.1 c	1.13 c	10.44 c	51.10 c	73.96 c	4.24 c	75.10 c	14.72 c
N120	405.4 b	248.7 b	1.21 b	12.57 b	55.01 b	81.14 b	4.75 b	80.66 b	15.89 b
N240	444.7 a	262.7 a	1.30 a	14.66 a	58.21 a	88.73 a	5.26 a	86.27 a	17.01 a
N360	434.6 a	254.5 ab	1.30 a	14.57 a	54.78 b	88.57 a	4.91 b	84.29 ab	17.03 a
Chemical regulation									
CK	390.12 b	233.6 b	1.16 b	12.28 b	51.97 b	78.45 b	4.49 b	77.39 b	15.34 b
PGR	436.63 a	265.3 a	1.31 a	13.84 a	57.58 a	87.75 a	5.09 a	85.77 a	16.98 a
Variety									
JNK728	427.4 a	257.3 a	1.27 a	13.51 a	56.36 a	85.44 a	4.93 a	84.22 a	16.64 a
SD5	399.4 b	241.7 b	1.20 b	12.62 b	53.19 b	80.76 b	4.65 b	78.94 b	15.68 b
Sources of variation									
V	**	**	**	**	**	**	**	**	**
N	**	**	**	**	**	**	**	**	**
C	**	**	**	**	**	**	**	**	**
V × N	NS	NS	NS	NS	NS	NS	NS	NS	NS
V × C	NS	NS	NS	*	NS	NS	NS	NS	NS
N × C	NS	NS	NS	NS	NS	NS	NS	NS	NS
V × N × C	NS	NS	NS	NS	NS	NS	NS	NS	NS
Milk stage									
Nitrogen application									
N0	138.0 d	118.5 c	0.596 c	6.39 d	26.18 c	44.87 c	1.94 c	31.32 c	4.28 c
N120	166.1 c	133.6 b	0.672 b	7.33 c	28.78 b	49.12 b	2.23 b	35.60 b	4.85 b
N240	207.3 a	144.4 a	0.746 a	8.36 a	31.13 a	54.39 a	2.55 a	40.28 a	5.83 a
N360	196.3 b	122.8 c	0.720 a	7.80 b	31.36 a	51.83 a	2.53 a	40.06 a	5.68 a
Chemical regulation									
CK	164.2 b	119.3 b	0.636 b	6.90 b	27.15 b	46.36 b	2.12 b	33.86 b	4.74 b
PGR	189.6 a	140.3 a	0.731 a	8.04 a	31.58 a	53.75 a	2.51 a	39.77 a	5.58 a
Variety									
JNK728	183.6 a	133.3 a	0.707 a	7.71 a	30.36 a	51.44 a	2.84 a	38.02 a	5.33 a
SD5	170.5 b	126.4 b	0.660 b	7.24 b	28.37 b	48.67 b	1.79 b	35.61 b	4.99 b
Sources of variation									
V	**	*	**	**	**	**	**	**	**
N	**	**	**	**	**	**	**	**	**
C	**	**	**	**	**	**	**	**	**
V × N	NS	NS	NS	NS	NS	NS	NS	NS	NS
V × C	NS	NS	NS	NS	NS	NS	NS	NS	NS
N × C	NS	NS	NS	NS	NS	NS	NS	NS	NS
V × N × C	NS	NS	NS	NS	NS	NS	NS	NS	NS

N0, N120, N240, and N360 indicate nitrogen application rates at 0, 120, 240, and 360 kg ha^−1^, respectively. PGR and CK indicate spraying plant growth regulator and water, respectively. JNK728 and SD5 indicate maize varieties Jingnongke 728 and Saide 5, respectively. V, N, and C indicate variety, nitrogen application, and chemical regulation, respectively. Values within a column for the same treatment followed by the different letters indicate a significant difference at *p* < 0.05. * and ** indicate significance at the 0.05 and 0.01 probability levels, respectively, and NS is not significant.

**Table 5:** Effect of nitrogen application rates and chemical regulation on amino acids concentrations in root bleeding sap (μg plant^−1^ h^−1^) (2022).

Treatment	Ser	Glu	Gly	Ala	Val	Lys	Met	Arg	Leu
Jointing stage									
Nitrogen application									
N0	399.0 d	292.6 c	1.25 c	12.89 d	55.13 c	89.96 c	4.66 c	81.42 c	15.92 c
N120	480.2 c	329.8 b	1.41 b	14.79 c	60.59 b	98.48 b	5.35 b	93.03 b	18.04 b
N240	599 a	356.4 a	1.57 a	16.86 a	65.56 a	109.1 a	6.14 a	103.84 a	21.67 a
N360	567.5 b	303.2 c	1.51 a	15.73 b	66.05 a	103.9 a	6.09 a	95.86 b	21.15 a
Chemical regulation									
CK	486.5 b	301.7 b	1.36 b	14.09 b	58.58 b	94.95 b	5.12 b	87.61 b	17.81 b
PGR	536.6 a	339.3 a	1.51 a	16.04 a	65.08 a	105.75 a	5.99 a	99.47 a	20.58 a
Variety									
JNK728	524 a	331.3 a	1.48 a	15.51 a	63.56 a	103.44 a	5.73 a	96.22 a	19.84 a
SD5	498.7 b	309.7 b	1.39 b	14.63 b	60.10 b	97.26 b	5.38 b	90.86 b	18.55 b
Sources of variation									
V	*	**	**	**	**	**	**	**	**
N	**	**	**	**	**	**	**	**	**
C	**	**	**	**	**	**	**	**	**
V × N	NS	NS	NS	NS	NS	NS	NS	NS	NS
V × C	*	NS	NS	NS	NS	NS	NS	NS	NS
N × C	NS	NS	NS	NS	NS	NS	NS	NS	NS
V × N × C	NS	NS	NS	NS	NS	NS	NS	NS	NS
Tasseling stage									
Nitrogen application									
N0	368.8 c	216.7 c	1.03 c	11.62 c	49.99 c	69.28 c	3.42 c	71.61 c	14.39 c
N120	396.5 b	234.8 b	1.14 b	12.61 b	50.63 b	77.03 b	4.19 b	76.27 b	15.30 b
N240	421.6 a	251.0 a	1.25 a	13.73 a	55.23 a	85.44 a	5.35 a	80.88 a	16.36 a
N360	406.3 ab	248.5 a	1.22 a	12.85 b	52.08 b	85.62 a	5.27 a	79.26 ab	15.61 ab
Chemical regulation									
CK	376.9 b	224.2 b	1.11 b	11.96 b	48.89 b	74.94 b	4.32 b	73.54 b	14.65 b
PGR	419.6 a	251.3 a	1.21 a	13.44 a	55.08 a	83.75 a	4.79 a	80.47 a	16.18 a
Variety									
JNK728	408.4 a	244.3 a	1.20 a	13.11 a	53.56 a	81.44 a	4.69 a	79.02 a	15.84 a
SD5	388.2 b	231.2 b	1.12 b	12.30 b	50.41 b	77.25 b	4.42 b	74.99 b	14.99 b
Sources of variation									
V	*	**	**	**	**	*	**	*	**
N	**	**	**	**	**	**	**	*	**
C	**	**	**	**	**	**	**	**	**
V × N	NS	NS	NS	NS	NS	NS	NS	NS	NS
V × C	NS	NS	NS	NS	NS	NS	NS	NS	NS
N × C	NS	NS	NS	NS	NS	NS	NS	NS	NS
V × N × C	NS	NS	NS	NS	NS	NS	NS	NS	NS
Milk stage									
Nitrogen application									
N0	114.5 c	111.8 c	0.579 c	5.82 c	23.07 d	36.03 c	1.78 c	28.33 c	4.34 d
N120	156.0 b	124.2 b	0.646 b	6.68 b	26.30 c	43.23 b	2.09 b	32.65 b	5.10 c
N240	211.1 a	135.8 a	0.721 a	7.88 a	29.96 a	55.17 a	2.39 a	37.16 a	5.87 a
N360	201.7 a	131.7 a	0.695 a	7.84 a	28.08 b	56.68 a	2.17 b	35.50 a	5.51 b
Chemical regulation									
CK	154.0 b	116.4 b	0.599 b	6.67 b	23.62 b	43.80 b	1.92 b	31.35 b	4.73 b
PGR	187.6 a	135.3 a	0.721 a	7.44 a	30.08 a	51.75 a	2.29 a	35.47 a	5.68 a
Variety									
JNK728	176.4 a	130.3 a	0.677 a	7.28 a	27.56 a	49.14 a	2.17 a	34.32 a	5.39 a
SD5	165.3	121.5	0.643	6.84	26.14	46.41	2.04	32.50	5.02
Sources of variation									
V	**	**	*	**	*	**	**	**	**
N	**	**	**	**	**	**	**	**	**
C	**	**	**	**	**	**	**	**	**
V × N	NS	NS	NS	NS	NS	NS	NS	NS	NS
V × C	NS	NS	NS	NS	NS	NS	NS	NS	NS
N × C	NS	NS	NS	NS	NS	NS	NS	NS	NS
V × N × C	NS	NS	NS	NS	NS	NS	NS	NS	NS

N0, N120, N240, and N360 indicate nitrogen application rates at 0, 120, 240, and 360 kg ha^−1^, respectively. PGR and CK indicate spraying plant growth regulator and water, respectively. JNK728 and SD5 indicate maize varieties Jingnongke 728 and Saide 5, respectively. V, N, and C indicate variety, nitrogen application, and chemical regulation, respectively. Values within a column for the same treatment followed by the different letters indicate a significant difference at *p* < 0.05. * and ** indicate significance at the 0.05 and 0.01 probability levels, respectively, and NS is not significant.

**Table 6:** Effect of nitrogen application rates and chemical regulation on grain-filling parameters.

Treatment	2021				2022			
	T_max_ (d)	V_max_ (g 100-grain^−1^ d^−1^)	V_m_ (g 100-grain^−1^ d^−1^)	P (d)	T_max_ (d)	V_max_ (g 100-grain^−1^ d^−1^)	V_m_ (g 100-grain^−1^ d^−1^)	P (d)
Nitrogen application								
N0	28.64 a	1.032 c	0.487 c	43.63 b	28.37 a	1.004 c	0.471 c	43.85 a
N120	29.33 a	1.097 b	0.519 b	45.58 ab	28.86 a	1.065 b	0.499 b	45.18 a
N240	29.69 a	1.165 a	0.554 a	46.95 a	29.23 a	1.129 a	0.530 a	45.92 a
N360	29.51 a	1.151 ab	0.545 a	46.07 a	29.02 a	1.112 ab	0.522 ab	45.56 a
Chemical regulation								
CK	29.75 a	1.083 b	0.511 b	45.44 a	29.29 a	1.049 b	0.492 b	45.35 a
PGR	28.84 a	1.14 a	0.542 a	45.68 a	28.45 a	1.106 a	0.519 a	44.91 a
Variety								
JNK728	30.42 a	1.081 b	0.512 b	46.32 a	29.75 a	1.049 b	0.493 b	45.74 a
SD5	28.17 b	1.142 a	0.541 a	44.80 a	27.99 b	1.106 a	0.518 a	44.52 a
Sources of variation								
V	**	**	**	NS	**	**	**	NS
N	NS	**	**	*	NS	**	**	NS
C	NS	**	**	NS	NS	**	**	NS
V × N	NS	NS	*	NS	NS	NS	NS	NS
V × C	NS	*	NS	NS	NS	NS	*	NS
N × C	NS	NS	NS	NS	NS	NS	NS	NS
V × N × C	NS	NS	NS	NS	NS	NS	NS	NS

T_max_: the time reaching the maximum grain-filling rate; V_max_: maximum grain-filling rate; V_m_: mean grain-filling rate; P: active grain-filling period. N0, N120, N240, and N360 indicate nitrogen application rates at 0, 120, 240, and 360 kg ha^−1^, respectively. PGR and CK indicate spraying plant growth regulator and water, respectively. JNK728 and SD5 indicate maize varieties Jingnongke 728 and Saide 5, respectively. V, N, and C indicate variety, nitrogen application, chemical regulation, respectively. Values within a column for the same treatment followed by the different letters indicate a significant difference at *p* < 0.05. * and ** indicate significance at the 0.05 and 0.01 probability levels, respectively, and NS is not significant.

**Table 7:** Effect of nitrogen application rates and chemical regulation on dry matter accumulation per plant of maize (2021).

Treatment	Dry Matter Accumulation per Plant (g plant^−1^)					ADMA	CPDMA
	Jointing Stage	Tasseling Stage	Early Filling Stage	Milk Stage	Maturity Stage	(g plant^−1^)	(%)
Nitrogen application							
N0	38.6 c	143.6 c	186.6 c	279.0 c	285.7 c	142.1 d	49.7 b
N120	43.9 b	155.9 b	202.0 b	306.2 b	330.9 b	175.0 c	52.9 a
N240	48.2 a	165.6 a	218.9 a	330.3 a	362.8 a	197.1 a	54.3 a
N360	49.7 a	165.1 a	217.2 a	320.6 ab	350.4 a	185.3 b	52.9 a
Chemical regulation							
CK	47.0 a	161.3 a	208.3 a	300.3 b	320.1 b	158.8 b	49.6 b
PGR	43.1 b	151.8 b	204.0 a	317.8 a	344.7 a	193.0 a	56.0 a
Variety							
JNK728	45.7 a	159.4 a	211.6 a	318.3 a	342.6 a	183.2 a	53.5 a
SD5	44.5 a	153.7 a	200.6 b	299.8 b	322.3 b	168.6 b	52.3 a
Sources of variation							
V	NS	NS	*	**	**	**	NS
N	**	**	**	**	**	**	*
C	**	*	NS	*	**	**	**
V × N	NS	NS	*	*	NS	NS	NS
V × C	NS	NS	*	NS	*	*	NS
N × C	NS	NS	NS	NS	NS	NS	NS
V × N × C	NS	NS	NS	NS	NS	NS	NS

N0, N120, N240, and N360 indicate nitrogen application rates at 0, 120, 240 and 360 kg ha^−1^, respectively. PGR and CK indicate spraying plant growth regulator and water, respectively. JNK728 and SD5 indicate maize varieties Jingnongke 728 and Saide 5, respectively. V, N, and C indicate variety, nitrogen application, and chemical regulation, respectively. Values within a column for the same treatment followed by the different letters indicate a significant difference at *p* < 0.05. * and ** indicate significance at the 0.05 and 0.01 probability levels, respectively, and NS is not significant.

**Table 8:** Effect of nitrogen application rates and chemical regulation on dry matter accumulation per plant of maize (2022).

Treatment	Dry Matter Accumulation per Plant (g plant^−1^)					ADMA	CPDMA
	Jointing Stage	Tasseling Stage	Early Filling Stage	Milk Stage	Maturity Stage	(g plant^−1^)	(%)
Nitrogen application							
N0	35.1 c	132.5 c	174.1 c	263.5 c	271.0 c	138.5 c	51.1 b
N120	39.0 b	139.4 b	185.7 b	281.6 b	303.0 b	163.5 b	54.0 a
N240	42.7 a	148.1 a	197.3 a	299.5 a	330.6 a	182.5 a	55.2 a
N360	44.3 a	150.4 a	196.9 a	297.3 a	329.1 a	178.7 a	54.3 a
Chemical regulation							
CK	46.4 a	161.9 a	210.3 a	277.2 b	297.0 b	135.2 b	45.5 b
PGR	42.1 b	151.5 b	204.0 a	293.8 a	319.8 a	168.2 a	52.6 a
Variety							
JNK728	44.8 a	159.9 a	213.0 a	325.6 a	320.1 a	160.1 a	50.0 a
SD5	43.7 a	153.5 a	201.3 b	305.1 b	296.7 b	143.3 b	48.3 a
Sources of variation							
V	NS	NS	**	**	**	**	NS
N	**	**	**	**	**	**	*
C	**	**	NS	**	**	**	**
V × N	NS	NS	NS	*	NS	NS	NS
V × C	NS	NS	*	NS	*	*	NS
N × C	NS	NS	*	NS	NS	NS	NS
V × N × C	NS	NS	NS	NS	NS	NS	NS

N0, N120, N240, and N360 indicate nitrogen application rates at 0, 120, 240 and 360 kg ha^−1^, respectively. PGR and CK indicate spraying plant growth regulator and water, respectively. JNK728 and SD5 indicate maize varieties Jingnongke 728 and Saide 5, respectively. V, N, and C indicate variety, nitrogen application, and chemical regulation, respectively. Values within a column for the same treatment followed by the different letters indicate a significant difference at *p* < 0.05. * and ** indicate significance at the 0.05 and 0.01 probability levels, respectively, and NS is not significant.

**Table 9:** Effect of nitrogen application rates and chemical regulation on yield and yield components.

Treatment	2021				2022			
	Yield (kg ha^−1^)	Ears Number (ears ha^−1^)	Grains Number per Ear	1000-Grain Weight (g)	Yield (kg ha^−1^)	Ears Number (ears ha^−1^)	Grains Number per Ear	1000-Grain Weight (g)
Nitrogen application								
N0	9748 c	81,162 a	415 c	358 b	9093 c	81,161 a	395 b	331 b
N120	11,350 b	81,280 a	442 b	366 ab	10,129 b	81,388 a	427 a	347 a
N240	11,724 a	81,386 a	457 a	376 a	10,560 a	81,413 a	433 a	355 a
N360	11,305 b	81,468 a	461 a	372 ab	10,421 a	81,444 a	428 a	351 a
Chemical regulation								
CK	10,409 b	81,213 a	425 b	359 b	9537 b	81,334 a	400 b	339 b
PGR	11,654 a	81,434 a	463 a	377 a	10,565 a	81,368 a	441 a	353 a
Variety								
JNK728	11,729 a	81,760 a	511 a	362 a	10,618 a	81,418 a	479 a	342 a
SD5	10,334 b	80,889 a	376 b	374 a	9484 b	81,285 a	362 b	351 a
Sources of variation								
V	**	NS	**	NS	**	NS	**	NS
N	**	NS	**	*	**	NS	**	**
C	**	NS	**	**	**	NS	**	**
V × N	*	NS	NS	NS	**	NS	NS	NS
V × C	*	NS	NS	NS	*	NS	NS	NS
N × C	NS	NS	NS	NS	*	NS	NS	NS
V × N × C	NS	NS	NS	*	NS	NS	NS	NS

N0, N120, N240, and N360 indicate nitrogen application rates at 0, 120, 240, and 360 kg ha^−1^, respectively. PGR and CK indicate spraying plant growth regulator and water, respectively. JNK728 and SD5 indicate maize varieties Jingnongke 728 and Saide 5, respectively. V, N, and C indicate variety, nitrogen application, and chemical regulation, respectively. Values within a column for the same treatment followed by the different letters indicate a significant difference at *p* < 0.05. * and ** indicate significance at the 0.05 and 0.01 probability levels, respectively, and NS is not significant.

**Table 10:** Meteorological data during 2021–2022 growing season of maize.

Year	Month	Average Temperature (°C)	Total Rainfall (mm)	Average Wind Velocity (m·s^−1^)
2021	May	16.08	80.77	6.01
	June	20.57	79.50	5.08
	July	25.96	167.89	4.55
	August	20.96	146.81	5.57
	September	16.20	63.75	4.12
2022	May	14.97	65.02	6.21
	June	20.84	128.02	5.44
	July	24.63	59.44	4.67
	August	20.96	187.71	4.55
	September	16.72	4.57	5.68

## Data Availability

Data are contained within the article.

## Abbreviations

The following abbreviations are used in this manuscript:

PGRs	Plant growth regulators
DA-6	Diethyl aminoethyl hexanoate
AGPase	ADP-glucose pyrophosphorylase
SSS	Soluble starch synthase
ADMA	Amount of dry matter per plant after anthesis
CPDMA	Contribution proportion of the dry matter after anthesis
GWP	Global warming potential
GHGI	Greenhouse gas intensity
